# Evaluation of the cost-effectiveness of the high-chloride vs. low-chloride crystalloid fluids in hospitalized patients from the us third-party provider perspective

**DOI:** 10.1186/2197-425X-3-S1-A156

**Published:** 2015-10-01

**Authors:** D Makhija, S Laplante, I Beer, C Schermer, L Perrault

**Affiliations:** Baxter Healthcare Corporation, Deerfield, United States; Former Baxter Healthcare Corporation Employee, Deerfield, United States; International Market Access Consulting, Zug, Switzerland

## Introduction

Numerous published studies identify acute kidney injury (AKI) as an independent risk factor for developing incident chronic kidney disease (CKD), progressive CKD, end-stage renal disease (ESRD), and other important non-renal outcomes with substantial public health implications. A recent meta-analysis showed that use of high-chloride crystalloids in critically ill patients was associated with a significantly higher risk of AKI (1).

## Objectives

The objective of this study was to evaluate the cost-effectiveness of using high-chloride vs. low-chloride crystalloid fluids in patients hospitalized in the intensive care unit (ICU) from the US third-party provider perspective.

## Methods

A Markov decision model was developed to assess the overall impact of chronic dialysis progression, both in survivors of AKI and in patients without AKI exposed to high-chloride vs. low-chloride crystalloids over the life time horizon. The model inputs, including costs, utilities, and probabilities, were extracted from the published literature. The relative risk of developing AKI was obtained from the meta-analysis of published randomized clinical trials and observational studies mentioned above (n= 9 studies with a total of 229 patients). The model simulated the patient outcomes, assessing the life years gained (LYG), quality-adjusted life years (QALYs), and associated health care costs.

## Results

The probability of patients developing AKI post fluid resuscitation with high-chloride crystalloids was 36% vs. 22% for patients receiving low-chloride crystalloids. At 90 days, the risk of patients progressing to chronic dialysis in AKI survivors receiving renal replacement therapy (RRT) was 88.04 per 100,000 patients in the high-chloride group vs. 53.68 per 100,000 patients in the low-chloride group. Overall costs were lower for patients receiving low-chloride crystalloids, translating into savings of $2,571 per patient over the lifetime horizon. The incremental cost-effectiveness ratio (ICER) of -$3,699 per QALY indicated that low-chloride crystalloids dominate (i.e. less costly and more effective) high-chloride crystalloids and was well below the societal willingness-to-pay threshold of $100,000 in the US. Various sensitivity analyses confirmed the robustness of these findings.

## Conclusions

In a Markov-based decision model of patients hospitalized in the intensive care unit, low-chloride crystalloids were dominant (less costly and more effective) compared to high-chloride crystalloids. The overall impact on patients progressing to chronic dialysis and the associated costs were significantly reduced in survivors of AKI receiving low-chloride fluid resuscitation.

## Grant Acknowledgment

This study was sponsored by Baxter.
